# Association between the Level of Enriched Environment and serum Brain-Derived Neurotrophic Factor (BDNF) in patients with Major Depressive Disorder

**DOI:** 10.1192/j.eurpsy.2025.735

**Published:** 2025-08-26

**Authors:** A. Vega-Rosas, M. Flores-Ramos, G. B. Ramírez-Rodríguez

**Affiliations:** 1 Subdivisión de Investigaciones Clínicas; 2 Laboratorio de Neurogénesis, Instituto Nacional de Psiquiatría Ramón de la Fuente Muñiz, Mexico City, Mexico

## Abstract

**Introduction:**

Major Depressive Disorder (MDD) is a neuropsychiatric condition whose neurobiological characteristics include alterations in brain plasticity, modulated by Brain Derived Neurotrophic Factor (BDNF). In animal models, Environmental Enrichment promotes neuroplasticity and reduces depressive-like behaviors. It has been proposed to measure the level of Enriched Environment (EE) as a protective or risk factor for the development and severity of MDD using the EE Indicator (EEI).

**Objectives:**

Determine the relationship between the level of EE and serum levels of BDNF in participants with MDD and healthy controls.

**Methods:**

Treatment-free MDD patients and controls were recruited, who underwent an analysis of their LES, clinical factors, and serum BDNF levels.

**Results:**

25 participants were recruited, of which 6 participants with MDD and the same number of controls were selected in a paired manner, who were divided into two groups:

medium and low EE. Although no differences were found between the concentration of BDNF between the groups, positive correlations were observed between social EE and BDNF, as well as negative correlations between this same domain with the Hamilton scale score for depression and the presence of this condition. No differences were found in the EE groups classified by total score between the cognitive, social and physical domains; But when breaking them down, it was observed that the sum between cognitive and social EE has a positive correlation with the serum concentration of BDNF (p=0.0451).

**Image 1:**

**Figure:**
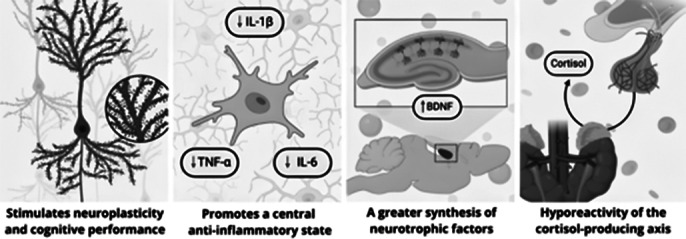

**Image 2:**

**Figure:**
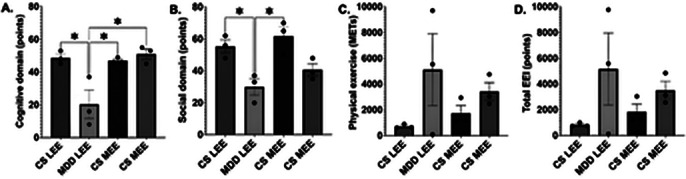

**Image 3:**

**Figure:**
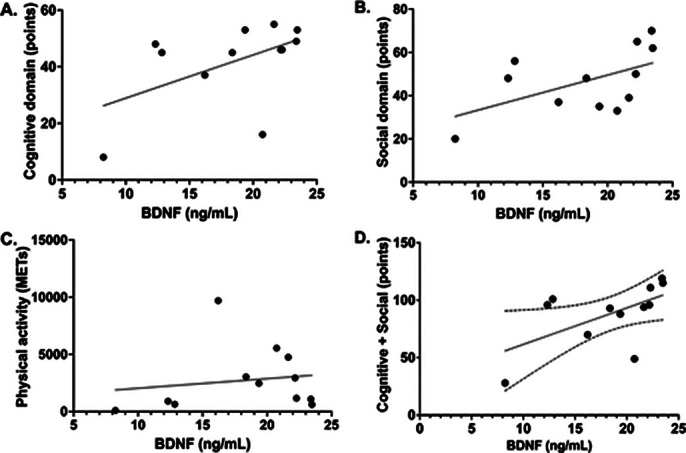

**Conclusions:**

The level of EE is potentially modulating the presence and severity of MDD at a clinical level, but it can also influence at a neuroplastic level through promoting or limiting the concentration of BDNF.

**Disclosure of Interest:**

None Declared

